# Development of a Recombination System for the Generation of Occlusion Positive Genetically Modified *Anticarsia Gemmatalis Multiple Nucleopolyhedrovirus*

**DOI:** 10.3390/v7041599

**Published:** 2015-03-31

**Authors:** Santiago Haase, Christina B. McCarthy, M. Leticia Ferrelli, Matias L. Pidre, Alicia Sciocco-Cap, Victor Romanowski

**Affiliations:** 1 Instituto de Biotecnología y Biología Molecular (IBBM), Departamento de Ciencias Biológicas, Facultad de Ciencias Exactas, Universidad Nacional de La Plata, CONICET, 1900-La Plata, Argentina; E-Mails: shaase@biol.unlp.edu.ar (S.H.); cmccarthy@conicet.gov.ar (C.B.M.); lferrelli@biol.unlp.edu.ar (M.L.F.); mlpidre@biol.unlp.edu.ar (M.L.P.); 2 Centro Regional de Estudios Genómicos (CREG), Facultad de Ciencias Exactas, Universidad Nacional de La Plata, 1900-La Platae, Argentina; 3 Instituto de Microbiología y Zoología Agrícola (IMYZA), Instituto Nacional de Tecnología Agropecuaria (INTA), 1712-Castelar, Argentina; E-Mail: sciocco.alicia@inta.gob.ar

**Keywords:** AgMNPV, recombinant baculovirus, bioinsecticide, *Anticarsia gemmatalis*, velvetbean caterpillar

## Abstract

*Anticarsia gemmatalis* is an important pest in legume crops in South America and it has been successfully controlled using *Anticarsia gemmatalis Multiple Nucleopolyhedrovirus* (AgMNPV) in subtropical climate zones. Nevertheless, in temperate climates its speed of kill is too slow. Taking this into account, genetic modification of AgMNPV could lead to improvements of its biopesticidal properties. Here we report the generation of a two-component system that allows the production of recombinant AgMNPV. This system is based on a parental AgMNPV in which the polyhedrin gene (*polh*) was replaced by a bacterial β*-galactosidase* (*lacZ*) gene flanked by two target sites for the homing endonuclease I-*Ppo*I. Co-transfection of insect cells with linearized (I-*Ppo*I-digested) parental genome and a transfer vector allowed the restitution of *polh* and the expression of a heterologous gene upon homologous recombination, with a low background of non-recombinant AgMNPV. The system was validated by constructing a recombinant occlusion-positive (*polh*^+^) AgMNPV expressing the green fluorescent protein gene (*gfp*). This recombinant virus infected larvae normally *per os* and led to the expression of GFP in cell culture as well as in *A. gemmatalis* larvae. These results demonstrate that the system is an efficient method for the generation of recombinant AgMNPV expressing heterologous genes, which can be used for manifold purposes, including biotechnological and pharmaceutical applications and the production of orally infectious recombinants with improved biopesticidal properties.

## 1. Introduction

Baculoviruses are double-stranded, circular DNA viruses that have drawn wide attention not only for their use as vectors for the expression of recombinant proteins in insect cells, but also for their potential use as biological control agents [1]. During virus replication, virions are included in large protein crystals called occlusion bodies that allow the virus to remain viable for many years in the environment, outside the insect host until another host ingests them. The velvetbean caterpillar*, Anticarsia gemmatalis* Hübner (*Lepidoptera*: *Noctuidae*) is considered one of the main foliage feeding pests of legume crops in South America, affecting mainly soybean fields. *Anticarsia gemmatalis multiple nucleopolyhedrovirus* (AgMNPV) belongs to the *Alphabaculovirus* genus of the *Baculoviridae* family and is used as a biological agent to control this pest, being the most widely used viral bioinsecticide worldwide [2,3,4].

However, a number of problems currently prevent the widespread use of this virus. A major drawback is that AgMNPV is relatively slow in killing its host insect in temperate climates. Depending on the age and susceptibility of the host, the virus may take up to two weeks to kill the insect. During that period, the insect can continue to feed. One approach to improve the speed of kill of baculoviruses is the introduction of foreign, insecticidal genes into the viral genome [5,6]. In order to facilitate the recovery of recombinant viruses expressing non-reporter genes, and to reduce the time required for recombinant virus cloning, the proportion of parental viruses in the progeny obtained after the co-transfection should ideally be suppressed. Several strategies have been developed for other baculoviruses (reviewed in [7]). One of the more exploited strategies to generate genetically modified baculoviruses relies on the homologous recombination mechanism. However, the frequency of recombination is low, and typically only 0.1%–2% of progeny viruses are recombinant. If linearized viral DNA, which cannot initiate a viral infection unless rescued by a recombination event, is used instead of circular wild type DNA, the proportion of recombinant progeny can be increased to about 30% [8]. Moreover, it was demonstrated that the incorporation of two endonuclease recognition sites in AcMNPV, was more effective in reducing the parental virus background progeny than the incorporation of a single unique site [9]. In this work we report the development of a system for the generation of AgMNPV recombinants consisting of two components: a parental occlusion negative baculovirus DNA engineered to contain the *E. coli lacZ* ORF flanked by two unique intron-encoded endonuclease sites used to linearize the viral DNA, and a transfer vector containing the *polyhedrin* (*polh*) gene and a heterologous gene of choice. After co-transfection of the linearized viral parental DNA with the transfer vector and recombination, the loss of the *lacZ* gene and the recovery of the polyhedrin gene are clear indicators that the recombinant virus has been generated.

## 2. Materials and Methods

### 2.1. Virus, Cells and Insects

AgMNPV-2D isolate (wild type) [10] was used as the parental virus for the generation of AgMNPV-I*-Ppo*I. UFLAg-286 (kindly supplied by Dr. Bergmann Ribeiro; Universidade de Brasília) and High-FiveTM (BTI-TN-5B1-4, Invitrogen^TM^, Carlsbad, CA, USA) cells were grown at 28 °C in TC-100 or Grace’s (Invitrogen^TM^) media containing 10% fetal bovine serum (Internegocios S.A., Mercedes, Argentina). *A. gemmatalis* were reared at IMYZA, (INTA, Castelar, Argentina). Larvae were maintained in the laboratory on an artificial diet [11] under controlled temperature (26 ± 1 °C), photoperiod (14L/10D) and 80% relative humidity.

### 2.2. Recombinant DNA Methods

The construction of vectors was performed according to standard molecular cloning procedures [12]. All constructs were confirmed by restriction analysis and sequencing. Oligonucleotide primers used in the study are indicated in Table 1 and Table 2 (see also Figure S1).

**Table 1:** Primers used to generate the transfer vectors pAg-I*Ppo*I and pI3. Relevant restriction sites incorporated in the primers are underlined and the nucleotides that anneal with the template are highlighted in bold.

Primer name	Primer sequence
Upr10-NdeI	GCCCATATG**CACAGTCAACGCCGGCC**
Lpr10-SgfI	GCCCGCGATCGC**GACGATATTGAAATGGTTGAAATAAATATAC**
Uprom-NdeI	GCCCCATATG**AAGTTGCAGCTCAAGCAGGATTGT**
Ppolhrev-NotI	CATTGCGGCCGCAATTCAAGCT**TAGTTATAGCAAATTTTACTAC**
Uup-RsrII	CCCCGGTCCG**ATGACCGAATTGAGCAACGCG**
Lup-SfiI	CTAGTTGGCCGCCTCGGC**C****TGCTGACTAAGCGTAGACC**
Lred-SfiI	CGCTTAGT GGCCGAGGCGGC**C****AACTAGAATGCAGTGAAAAAAATG**
SV40/CcdB-XmaI	ATGGACCACCCCGG**G****TTCCTGTAGCGGCCGCG**
Polhi-SgfI	AAATTTGCGATCG**C****TATGCCAGATTATACG**
Ldw-BglII	GGAAAGATCTATACACACGTTAG**GCGAGCGCCG**
eGFP/Up-EcoRI	TCCATCGAATT**C****ATGGTGAGCAAGGGC**
eGFP/Dw-XhoI	CTGATAAGCTTCTC**GAG****TCGCGGCCG**

**Table 2:** Primers used for the characterization of cloned AgMNPV-GFP. The nucleotides that anneal with the template are highlighted in bold. Relevant restriction sites incorporated in the primers are underlined.

Primer name	Primer sequence
Polhi-SgfI	AAATTTGCGATCGCT**ATGCCAGATTATACG**
AgDwrec	**AACCCGTAAAGCCGCCGTTG**
AgUpsrec	**GGCGCGAGTTAAATAGTCTG**
SV40/CcdB-XmaI	ATGGACCACCCCGG**G****TTCCTGTAGCGGCCGCG**
LacZ	**TGGATCTGCAACATGTCCCAGGTGA**
ppolhAg600UpHindIII	TGTACAAAGCT**T****CTAATTGCGTAAAAATG**
pie1Agfw	TATAAGATCTC**AGGGTACAATTG**
pie1Agrev	CATGAAGA**TCTATTTATACC**

### 2.3. Construction of the AgMNPV-I-PpoI Recombinant

**Figure 1:**
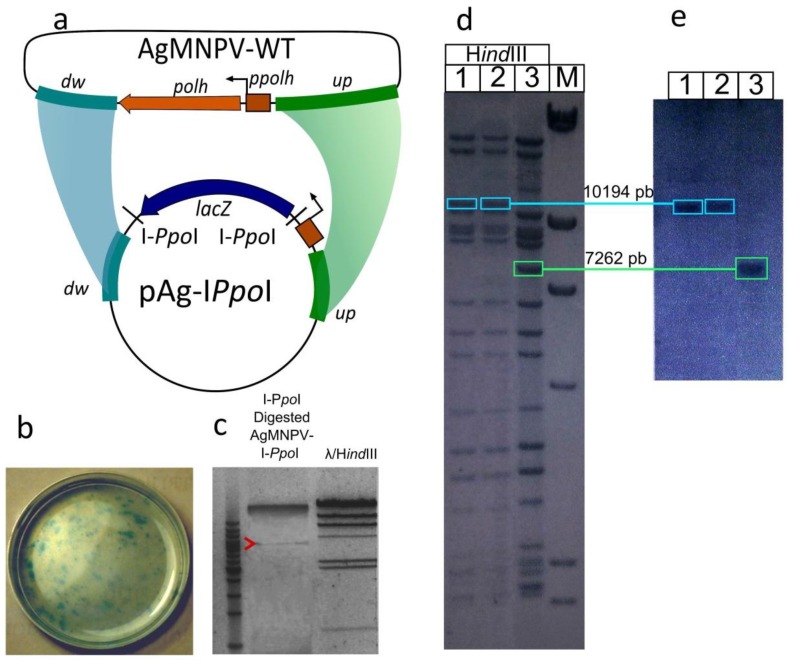
Generation of the parental genome AgMNPV-I*-Ppo*I. (**a**) Homologous recombination between AgMNPV-2D and pAgI*Ppo*I substitutes the *lacZ* ORF for the polyhedrin ORF and provides two I-*Ppo*I restriction sites. The sequences flanking the *polh* gene are indicated as *up* (upstream: 829 bp) and *dw* (downstream: 883 bp); (**b**) Cell culture dish infected with AgMNPV**-**I*-Ppo*I showing lacZ β-galactosidase-positive plaques; (**c**) AgMNPV-I*-Ppo*I digested with I-*Ppo*I: release of a 3 kb fragment (see arrowhead) containing the *lacZ* ORF flanked by I*-Ppo*I restriction sites; (**d**) *Hin*dIII restriction patterns of genomic DNA from two AgMNPV-I*-Ppo*I clones (lanes 1, 2) and wt AgMNPV (lane 3). The *Hin*dIII G bands are highlighted with boxes; (**e**) Southern blot using a probe targeting downstream *polh* sequences in band G with (10,194 bp; lanes 1, 2) or without (7262 bp) the *lacZ* ORF (lane 3).

Transfer vector pAg-I*-Ppo*I was constructed as described by McCarthy and Romanowski [13] (Figure 1). Briefly, linkers containing the I*-Ppo*I recognition sequence (CTCTCTTAA'GGTAGC) were introduced flanking the *lacZ* gene in pAgPHZ [14]. AgMNPV-2D DNA was then co-transfected with pAg-I*Ppo*I in UFLAg-286 cells using Tfx-20™ (Promega, Fitchburg, WI, USA) (Figure 1). Recombinant AgMNPV-I*-Ppo*I was isolated by five successive rounds of plaque purification and a simplified method for the extraction of baculoviral DNA for PCR analysis was employed to test the viruses each step [15]. Finally, the AgMNPV-I*-Ppo*I was amplified and its genomic DNA was purified from cell culture supernatant according to standard protocols [16]. The identity of the recombinant virus was confirmed by PCR, restriction analysis and Southern blot hybridization (Figure 1). I*-Ppo*I digestion conditions were previously optimized in our laboratory in order to achieve complete digestion of the recognition sequence embedded in different contexts [13].

### 2.4. Transfection of Linearized AgMNPV-I-PpoI DNA vs. Circular Undigested Genome

UFLAg-286 cells were transfected with linearized and undigested AgMNPV-I-*Ppo*I DNAs using Tfx-20™ (Promega, Fitchburg, WI, USA), according to the manufacturer’s protocol (500 ng/35 mm dish). I*-Ppo*I digestion conditions were optimized as descripted previously [13]. Five days after transfection, the cell culture supernatant was replaced with 3.5 mL Grace’s medium containing 10% fetal bovine serum (Internegocios S.A., Mercedes, Buenos Aires, Argentina) and 120 μg/mL X-Gal (5-bromo-4-chloro-3-indolyl-β-d-galacto-pyranoside) (Sigma-Aldrich^®^, St. Louis, MO, USA). One day later, cells were washed twice with phosphate buffered saline (PBS) and harvested in 1 mL lysis buffer (0.25% SDS, 10 mM Tris-HCl pH 7.6). Cell lysates were clarified by centrifugation at 1000× *g* for 10 min and 50 μL of supernatant was measured in a spectrometer (spectra were recorded for the 400–700 nm wavelength range). Relative enzyme activity was measured as units of absorbance at 650 nm—the maximum absorbance wavelength for blue-colored X-Gal hydrolysis product (colorless galactose plus 5-bromo-4-chloro-3-hydroxyindole, which spontaneously dimerizes and is oxidized into 5,5'-dibromo-4,4'-dichloro-indigo).

### 2.5. Generation of the Transfer Vector pI3

Transfer vector pI3 was constructed by sequential ligation of fragments from the AgMNPV-2D genome and fragments derived from commercial vectors (Figure S1). The primers used for the generation of the plasmid are listed in Table 1. In the first stage, a fragment containing the promoters of the *p10* and *polyhedrin* (*polh*) genes in opposite orientation was constructed following a combination of PCR amplifications, restriction digestions and ligations, as described in the following lines. The *p10* promoter was amplified with primers Upr10-*Nde*I and ligated in pGEM-T-Easy vector (generating pGEM-T-pp10). The *polh* promoter was amplified with primers Uprom-NdeI and pPolhrev-NotI. The *polh* promoter PCR product and the pGEM-T-pp10 vector were digested with *Nde*I and *Pst*I (*Pst*I site present in pGEM-T-Easy vector) and ligated in the *Nhe*I and *Pst*I sites from, pGEM-T-pp10, generating pGEM-T-pp10-ppolh. The upstream region of the polyhedrin gene was amplified by PCR; the SV40 polyadenylation signal was added by Splice Overlap Extension (SOE) PCR and ligated in the *Rsr*II/*Not*I digested pIRES plasmid (Clontech), generating the pIRES-UpspA vector. The primers used to amplify the upstream region were Uup-*Rsr* and Lup-*Sfi*, and the SV40 polyA was amplified from the commercial vector pDsRed1-N1 (Clontech) with primers Lred-*Sfi* and SV40/CcdB-*Xma*I. The fragment containing the *p10* promoter and the *polh* promoter in a back-to-back orientation was first released from pGEM-T-p10-ppolh by digestion with *Not*I and then ligated in the same restriction site of pIRESUpspA, yielding the pIRES-UpspA-pp10-ppolh vector. Finally, the *polh* ORF sequence with the *polh* gene downstream region was amplified from the AgMNPV-2D genome with primers Polhi-*Sgf*I and Ldw-*Bgl*II and ligated in pIRES-UpspA-pp10-ppolh S*gf*I and *Bgl*II recognition sites, generating transfer vector pI3 (Figure S1).

### 2.6. Construction of Recombinant AgMNPV-GFP

Enhanced GFP ORF was amplified by PCR from the commercial vector peGFP-N3 (Clontech) with primers eGFP/Up-*Eco*RI and eGFP/Down-*Xho*I, and cloned in pGEM-T-Easy (Invitrogen) following the manufacturer’s recommendations. The plasmid pI3-GFP was then constructed by releasing the GFP ORF from pGEM-T-GFP by digestion with *EcoR*I and subsequently ligating it into *EcoR*I-digested and dephosphorylated pI3.

**Figure 2:**
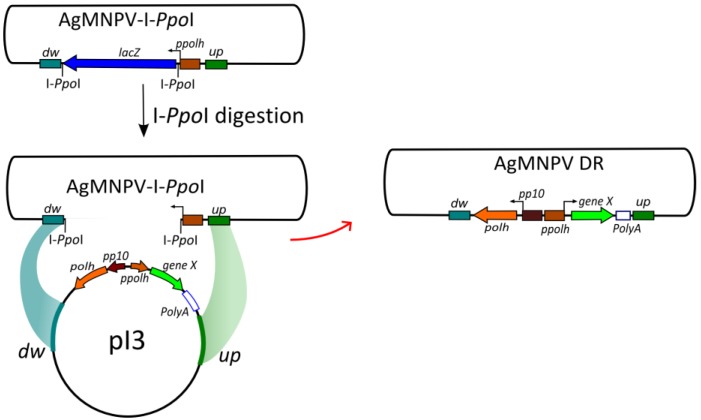
Schematic representation of the recombination system. Parental genome (AgMNPV-I*-Ppo*I) is linearized by digestion with I*-Ppo*I, and co-transfected with the transfer vector (pI3). Homologous recombination restores genome viability (re-circularization) generating recombinant progeny. Sequences flanking the original *polh* gene, where recombination can occur, are indicated as *dw* (511 bp downstream *polh* ORF stop codon; AgMNPV genome nucleotide positions 132,239–131,729) and *up* (610 bp upstream the *polh* promoter; AgMNPV genome nucleotides positions 934–1543). See [4] for AgMNPV genome nucleotide numbers.

**Figure 3:**
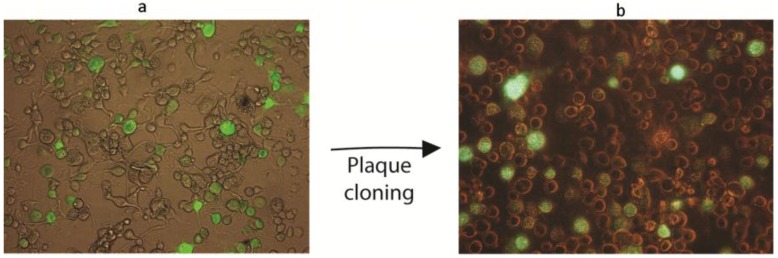
Generation of AgMNPV-GFP. (**a**) GFP (green fluorescent protein) expression in High Five™ cells co-transfected with linearized AgMNPV-I*-*P*po*I and pI3-GFP (schematic in Figure 4a); (**b**) High Five™ cells infected with cloned AgMNPV-GFP. Cells were infected with recombinant virus recovered from the plug of the agarose overlay from a single selected plaque. The differences in the levels of GFP expression of individual cells are related to the low multiplicity of infection.

In order to generate AgMNPV-GFP, High Five^TM^ cells were co-transfected with 2 μg of linearized parental AgMNPV-I*-Ppo*I viral DNA and 2 μg of pI3-GFP using Cellfectin II™ (Invitrogen™), according to the manufacturer’s recommendations (Figure 2 and Figure 3).

**Figure 4:**
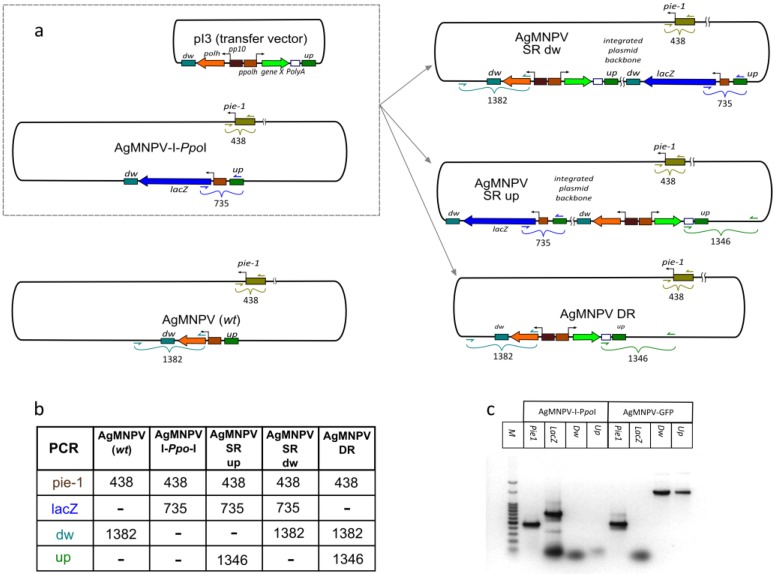
Characterization of cloned AgMNPV-GFP. (**a**) Schematic representation of plasmid (pI3-gene X) and viral DNA (AgMNPV-I-*Ppo*I) used in the study and possible viral genomic recombination products in plasmid-genome cotransfections. Genomes that could arise from uncut AgMNPV-I-*Ppo*I DNA are also included (SR: Single Recombinant; DR: Double Recombinant; *dw* and *up*: downstream and upstream *polh* ORF sequences where recombination could occur) Positions of PCR primers are indicated. A scheme of a wt AgMNPV is also included in order to clarify the origin of the putative PCR products that are shown in the table. (**b**) Table presenting the sizes of expected PCR amplicons in each possible genomic product (primers are those listed in Table 2); (**c**) Agarose gel electrophoretic patterns of PCR products using cloned AgMNPV-GFP (DR) and AgMNPV-I*-Ppo*I DNAs as templates.

When occlusion bodies and GFP fluorescence became apparent in the transfected cells, the supernatant was collected and used to infect High Five™ cell monolayers for subsequent plaque purification [17]. Occlusion-positive/GFP-positive plaques were selected and purified through three successive rounds of plaque isolation.

### 2.7. Characterization of AgMNPV-GFP

AgMNPV-GFP was amplified in High Five™ cells and the infected cell monolayers were observed by fluorescence microscopy (Nikon Eclipse Ti, Tokyo, Japan). AgMNPV-GFP and AgMNPV-I*-Ppo*I genomic DNA were purified from cell culture supernatants according to standard protocols [17]. The primers used for the characterization of the AgMNPV-GFP recombinant are listed in Table 2. The primers polhi-*Sgf*I and AgDwrec were used to detect a recombination in the *polh* downstream sequence; to detect recombination in the *polh* upstream region, the primers were AgUpsrec and SV40/CcdB-*Xma*I; and to detect the *lacZ* ORF in parental undigested DNA and simple homologous recombinants, the primers used were LacZ and ppolhAg600Up*Hin*dIII. The upstream and downstream primers anneal to AgMNPV sequences flanking the recombination region but not to transfer plasmid sequences. Consequently, an amplification fragment of the expected size is observed only when homologous recombination has taken place. A PCR amplification product of the *ie1* gene promoter was used as a positive control for AgMNPV DNA template, using primers pie1Agfw and pie1Agrev.

### 2.8. Per os Infection of A. gemmatalis Larvae with Recombinant AgMNPV

AgMNPV-GFP and AgMNPV-wt polyhedra were generated in High Five™ cells and purified according to standard protocols [16]. Polyhedra suspensions were prepared in sterile distilled water containing 1% (*v*/*v*) Coomassie brilliant blue (Sigma-Aldrich^®^, St. Louis, MO, USA) and sucrose (Sigma-Aldrich^®^). *A. gemmatalis* 3rd instar larvae were fed with this preparation using a modified droplet feeding method [18]. Larvae were infected with 2000 OB/larva (approximately 10 LD50), taking into account the previously reported mean volume ingested by *A. gemmatalis* 3rd instar larvae [19] and LD_50_ data for larvae from this same instar [20]. Infected larvae were evidenced by the presence of fluorescence when whole organisms were exposed to UV light. After five days post-infection, larvae were dissected and hemolymph cells and tracheoles were observed using fluorescence microscopy (Nikon Eclipse Ti).

## 3. Results

### 3.1. Construction of a Linearizable AgMNPV Genome

In order to simplify the isolation of genetically modified AgMNPVs, a homologous recombination system for AgMNPV based on the linearization of the parental genome was developed (Figure 1). The parental genome, designated AgMNPV-I*-Ppo*I, was generated by homologous recombination between AgMNPV-2D (referred to as wt, Zanotto *et al.* 1992) and pAg-I-*Ppo*I (*lacZ*^+^) transfer vector after co-transfection of an insect cell culture (see Materials and Methods). Virus plaques with a *lacZ*^+^ *polh*-phenotype were identified (Figure 2b) and isolated. In AgMNPV-I-*Ppo*I the polyhedrin ORF was replaced with the *E. coli* β*-galactosidase* ORF (*lacZ*) flanked by two sites of recognition for the intron-encoded endonuclease I-*Ppo*I, which enable the linearization of the genome.

AgMNPV-I*-Ppo*I DNA was characterized by restriction analysis and Southern blot (Figure 2). AgMNPV-wt and AgMNPV-I*-Ppo*I *Hin*dIII restriction patterns were differentiated by a change in the G band, from 7262 bp in AgMNPV-wt to 10,194 bp in AgMNPV-I*-Ppo*I. Also, after digesting AgMNPV-I*-Ppo*I with I-*Ppo*I, a 3 kb fragment (containing the *lacZ* ORF) was detected (Figure 2).

### 3.2. Transfection of Insect Cells with Circular vs. Linear AgMNPV Genomic DNA

In order to demonstrate the efficacy of the linearization of AgMNPV-I*-Ppo*I DNA in reducing parental genome viability, LacZ expression was assessed in cell monolayers transfected in parallel with circular and I-*Ppo*I-digested viral DNA. As described in Materials and Methods, the relative enzyme activity was estimated by measuring the hydrolysis of X-Gal: a decrease of *ca.* 40-fold in hydrolyzed X-Gal, was observed in cells transfected with linearized parental genome when compared with non-digested genomic DNA (data not shown), indicating that linearization was not 100% complete, but significantly lowered the amount of parental infectious virus recovered in the transfection.

### 3.3. Construction of a Transfer Vector for the Generation of Occlusion-Positive Recombinant AgMNPV

The transfer vector pI3 was constructed in order to introduce genetic modifications in the *polh* locus of AgMNPV. The salient features are two strong AgMNPV promoters (*polh* and *p10*) placed back to back. This cassette is flanked by upstream and downstream sequences surrounding the *polh* ORF in the AgMNPV genome (details can be found in Section 2.5 and supplementary file). Since the final aim was to produce recombinant AgMNPVs orally infectious (*polh*^+^) to *A. gemmatalis* larvae, pI3 was designed to contain the *polh* ORF (preceded by p10 5'-untranslated sequence) under the control of the *p10* promoter and followed by the *polh* downstream region, including the transcriptional termination signal. Three unique restriction sites placed after the *polh* promoter can be used to clone the ORF of choice, followed by the SV40 polyadenylation-termination signal and the *polh* upstream region (Figure 2). The transfer plasmid pI3-GFP was constructed by ligating *gfp* ORF in pI3 previously digested with *Eco*RI (Section 2.6).

High Five™ cells were cotransfected with pI3 and linearized (digested) or circular (undigested) AgMNPV-I-*Ppo*I. Supernatants of these transfections were used to infect High Five™ monolayers in varying dilutions and the proportions of white (recombinant) and blue (parental) plaques was evaluated using standard protocols with chromogenic β-galactosidase substrate X-Gal. Transfection using circular parental genomic DNA yielded more than 99% of blue and less than 1% white plaques. In contrast, when digested AgMNPV-I-*Ppo*I DNA was cotransfected with pI3, 90% of the observed plaques were white and only 10% were blue (data not shown).

### 3.4. Co-Transfection of Viral DNA and Transfer Plasmid

After the generation of the parental genome (AgMNPV-I*-Ppo*I), a recombinant AgMNPV carrying the *gfp* gene under the control of the polyhedrin promoter was generated in order to validate the recombination system (Figure 3). AgMNPV-GFP was produced by homologous recombination between linearized AgMNPV-I*-Ppo*I and the transfer vector pI3-GFP. The virus was cloned by three rounds of plaque purification. A cell monolayer was infected with cloned AgMNPV-GFP, and GFP expression and production of occlusion bodies were verified (Figure 3).

The viral DNA was characterized by PCR using the sets of primers mentioned in Materials and Methods (Table 2). The results indicate that the AgMNPV-GFP clone analyzed was derived from double homologous recombination between AgMNPV-I*-Ppo*I and pI3-GFP (Figure 4). In this process, *lacZ* was replaced with a cassette containing *polh* and *gfp* genes under the control of very late baculoviral promoters.

### 3.5. The Recombinant AgMNPV-GFP Infects Cells Producing Polyhedra, which Are Orally Infective to A. gemmatalis Larvae

**Figure 5:**
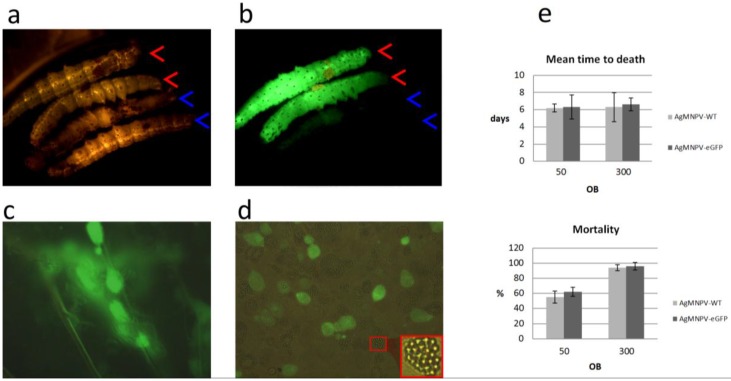
*A. gemmatalis* larvae orally infected with AgMNPV-GFP OBs (red arrowheads) and wt AgMNPV OBs (blue arrowheads) exposed to visible light (**a**) and UV light (**b**); tracheolar (**c**) and hemolymph (**d**) cells extracted from *A. gemmatalis* larvae infected with AgMNPV-GFP OBs. In the inset panel, polyhedra inside the nucleus of an infected cell are highlighted. (**e**) Results of mean time to death and mortality of *A. gemmatalis* larvae infected with recombinant AgMNPV-GFP and wt AgMNPV (*n* = 20 per treatment). The larvae consumed 50 (1 LD_50_) and 300 OBs (6 LD_50_) in parallel experiments.

After assessing the genetic organization of plaque-cloned AgMNPV-GFP and observing its ability to express both GFP and polyhedrin and to generate occlusion bodies in insect cell culture (Figure 3b), infectivity via oral route was evaluated on *A. gemmatalis* larvae (Figure 5). No differences in insecticidal parameters were evidenced when compared with wild type virus (Figure 5). GFP expression in whole animal and in secondary tissues of larvae infected with AgMNPV-GFP was observed (Figure 5), demonstrating the dispersion of the infection in the larvae.

## 4. Discussion

A limited number of recombinant AgMNPV have been reported and used to study the biology of this virus or to improve its biopesticidal parameters [14,21,22,23,24]. Recently, the generation of an occlusion-positive (*polh*^+^) recombinant AgMNPV carrying CfDefNPV v-*cath* (cathepsin) and *chiA* (chitinase A) genes was reported [21]. This recombinant AgMNPV showed an improved insecticidal activity against *A. gemmatalis* larvae when compared with AgMNPV-2D. However, owing to the lack of an efficient recombination system, the isolation of the recombinant virus demanded seven isolation cycles. On the other hand, the genetic characterization of this recombinant AgMNPV does not allow distinguishing if it is the result of double or simple homologous recombination. Considering that it takes about 10 days to complete one round of plaque isolation, the generation of recombinant viruses requires at least three months. In this regard, it has been previously reported that serial passages of recombinant baculoviruses in cell culture must be minimized because they may lead to the accumulation of defective viruses that can become the predominant species produced [25]. This is of special importance when a recombinant virus is to be tested as bioinsecticide, since the accumulation of mutations in genes related to oral infectivity does not affect virus propagation in cell culture. Furthermore, since the simultaneous manipulation of multiple recombinant baculovirus is not recommended because of the risk of cross-contamination, the time required for genetically modified baculovirus cloning limits the number of alternative recombinants that can be obtained.

As mentioned previously, the classical way of making recombinant viruses is based on homologous recombination between the viral genome and a transfer plasmid in insect cells, where the frequency of recombination is low, and only 0.1%–2% of progeny viruses are recombinant. In this respect, earlier reports showed that linearization of the baculovirus DNA at a single unique site increased the proportion of recombinant viruses to about 30% [8], whereas the incorporation of two unique restriction sites resulted in recombination frequencies of over 90% [9]. Given that linearized viral DNA was much less infectious than the circular form, this resulted in a great reduction in the background of non-recombinant viruses, but a relatively small reduction in the yield of recombinant viruses was observed [9]. Furthermore, the high proportion of recombinants following transfection with linear viral DNA was due to a reduction in the background of parental viruses, rather than to an increase in the absolute number of recombinants obtained. However, the presence of a small proportion of uncut viral-parental DNA cannot be completely ruled out and might be the source of unwanted non-recombinant progeny and might also yield single cross-over recombinants (SR genomes in Figure 4) and aberrant non-homologous recombination products. In our hands, a single round of isolated plaque purification is sufficient to assure a pure clone of rAgMNPV, e.g., AgMNPV-*polh*^+^*gfp*^+^. Two additional plaque purification rounds were done to completely rule out the carryover of contaminant genotypes.

Initial experiments were conducted using UFLAg-286 cells, which are highly efficient for AgMNPV replication; however, culture and lipid-mediated transfection of High Five™ cells turned out to be easier in our hands without seriously compromising virus yields. Therefore, this cell line is currently used in our lab for genetic modifications of AgMNPV.

In summary, a versatile recombination system for the efficient genetic modification of AgMNPV was developed and tested. This two-component system is based on the linearization of a parental baculovirus DNA and a transfer vector containing the *polyhedrin* gene and a heterologous gene of choice (both under the control of very late viral strong promoters). Our results prove that the recombination system described here significantly reduces the amount of time required for clonal purification of genetically modified AgMNPV. To validate the procedure, a recombinant AgMNPV harboring the *gfp* gene was produced; this virus efficiently infected *A. gemmatalis* larvae by oral route and expressed the heterologous gene at high levels.

Pest control using wt AgMNPV has been very successful in Brazil in the past 30 years, but failed in more temperate climates because of the relatively slow speed of kill of the virus in these conditions. The two-component system described here will facilitate the generation of AgMNPV recombinants, including heterologous genes designed to improve its bioinsecticidal capacity or to address other aspects of fundamental or applied baculovirus molecular biology.

## Supplementary Files

Supplementary File 1
