# Supplementary material for: Development of a Recombination System for the Generation of Occlusion Positive Genetically Modified Anticarsia Gemmatalis Multiple Nucleopolyhedrovirus
Source: Viruses. 2015 Mar 31;7(4):1599–612. doi: 10.3390/v7041599 (PMC4411667; doi:10.3390/v7041599)
Supplement: Supplementary File 1 [file viruses-07-01599-s001.pdf]

Supplementary Information

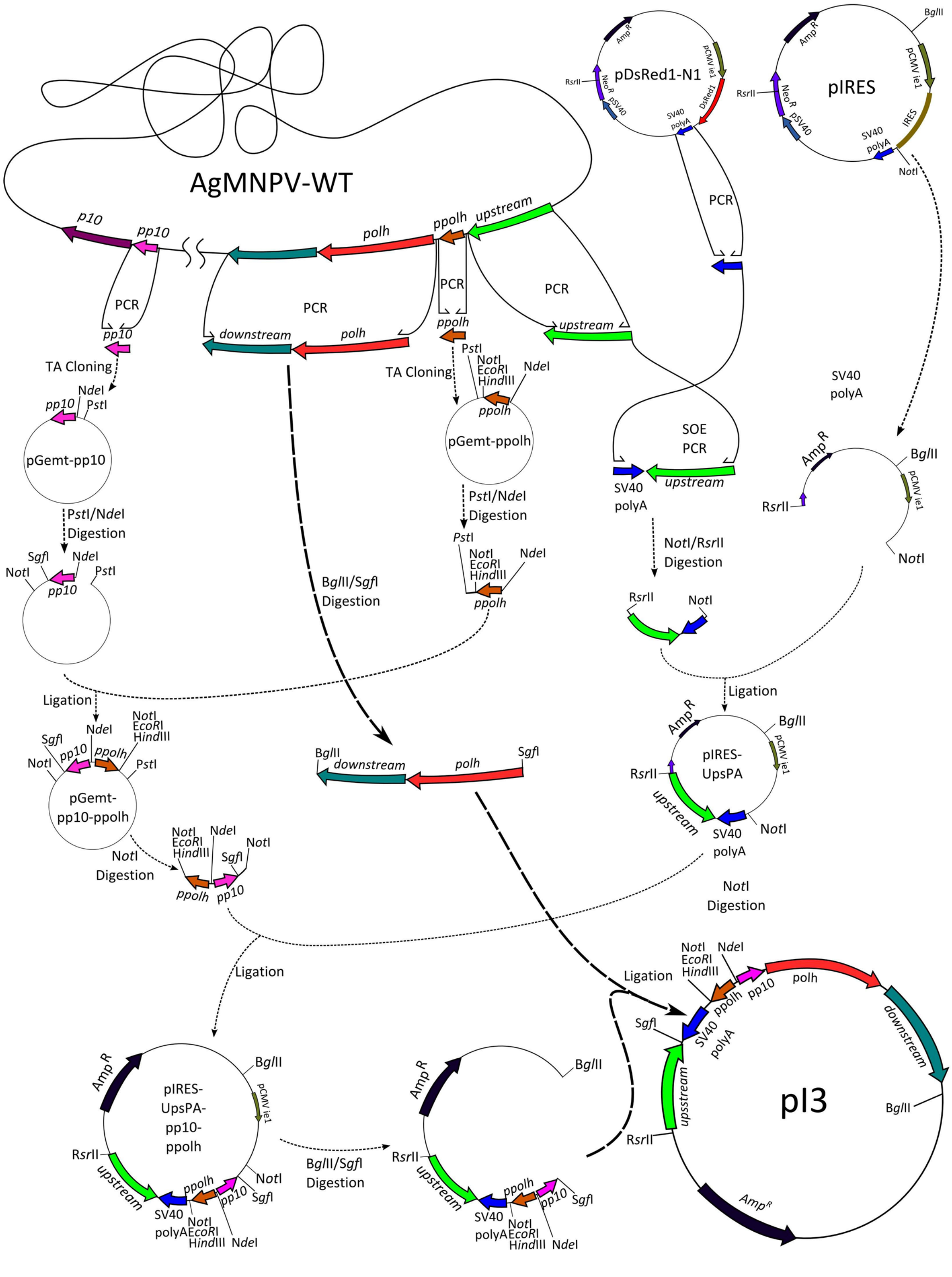

Figure S1. Schematic representation of the construction of pI3 transfer vector. Details provided in Materials and Methods section.
